# Monoclonal antibodies targeting the influenza virus N6 neuraminidase

**DOI:** 10.3389/fimmu.2022.944907

**Published:** 2022-07-27

**Authors:** Shirin Strohmeier, Fatima Amanat, Juan Manuel Carreño, Florian Krammer

**Affiliations:** ^1^ Department of Microbiology, Icahn School of Medicine at Mount Sinai, New York, NY, United States; ^2^ Department of Biotechnology, University of Natural Resources and Life Sciences, Vienna, Austria; ^3^ Graduate School of Biomedical Sciences, Icahn School of Medicine at Mount Sinai, New York, NY, United States; ^4^ Center for Vaccine Research and Pandemic Preparedness (C-VARPP), Icahn School of Medicine at Mount Sinai, New York, NY, United States; ^5^ Department of Pathology, Molecular and Cell Based Medicine, Icahn School of Medicine at Mount Sinai, New York, NY, United States

**Keywords:** influenza, neuraminidase, antibodies, N6, H4N6

## Abstract

Influenza A viruses are a diverse species that include 16 true hemagglutinin (HA) subtypes and 9 true neuraminidase (NA) subtypes. While the antigenicity of many HA subtypes is reasonably well studied, less is known about NA antigenicity, especially when it comes to non-human subtypes that only circulate in animal reservoirs. The N6 subtype NAs are mostly found in viruses infecting birds. However, they have also been identified in viruses that infect mammals, such as swine and seals. More recently, highly pathogenic H5N6 subtype viruses have caused rare infections and mortality in humans. Here, we generated murine mAbs to the N6 NA, characterized their breadth and antiviral properties *in vitro* and *in vivo* and mapped their epitopes by generating escape mutant viruses. We found that the antibodies had broad reactivity across the American and Eurasian N6 lineages, but relatively little binding to the H5N6 NA. Several of the antibodies exhibited strong NA inhibition activity and some also showed activity in the antibody dependent cellular cytotoxicity reporter assay and neutralization assay. In addition, we generated escape mutant viruses for six monoclonal antibodies and found mutations on the lateral ridge of the NA. Lastly, we observed variable protection in H4N6 mouse challenge models when the antibodies were given prophylactically.

## Introduction

Influenza A viruses are categorized based on 16 true hemagglutinin (HAs) subtypes and 9 true neuraminidase (NAs) subtypes which are all found in the avian reservoir (1). Two HA-like and NA-like surface glycoproteins have also been found in influenza-like viruses in bats (2, 3). HAs facilitate binding and entry into host cells and immune responses to HA have been correlated with protection from infection and/or disease. As such, the antigenicity of numerous HAs is highly characterized. However, NA is also very important in the viral life cycle. Its enzymatic activity cleaves terminal sialic acids (the receptor to which HA binds) from N-linked glycans. This activity allows incoming virus to avoid being trapped by highly sialilated natural defense proteins like mucins on mucosal surfaces (4, 5) and it is essential for release of budding virus from infected cells (6). NA-based immunity has been described as infection-permissive but can protect from disease and has been established as independent correlate of protection in humans (7, 8). Despite increased efforts to better understand NA antigenicity of human seasonal N1, N2 and type B NAs (9–21) for the design of improved influenza virus vaccines, little work has been done on non-human NA subtypes (group 1: N1, N4, N5, N8; group 2: N2, N3, N6, N7, N9), and most work has focused almost exclusively on the N9 NA (22–27).

**Figure 1:**
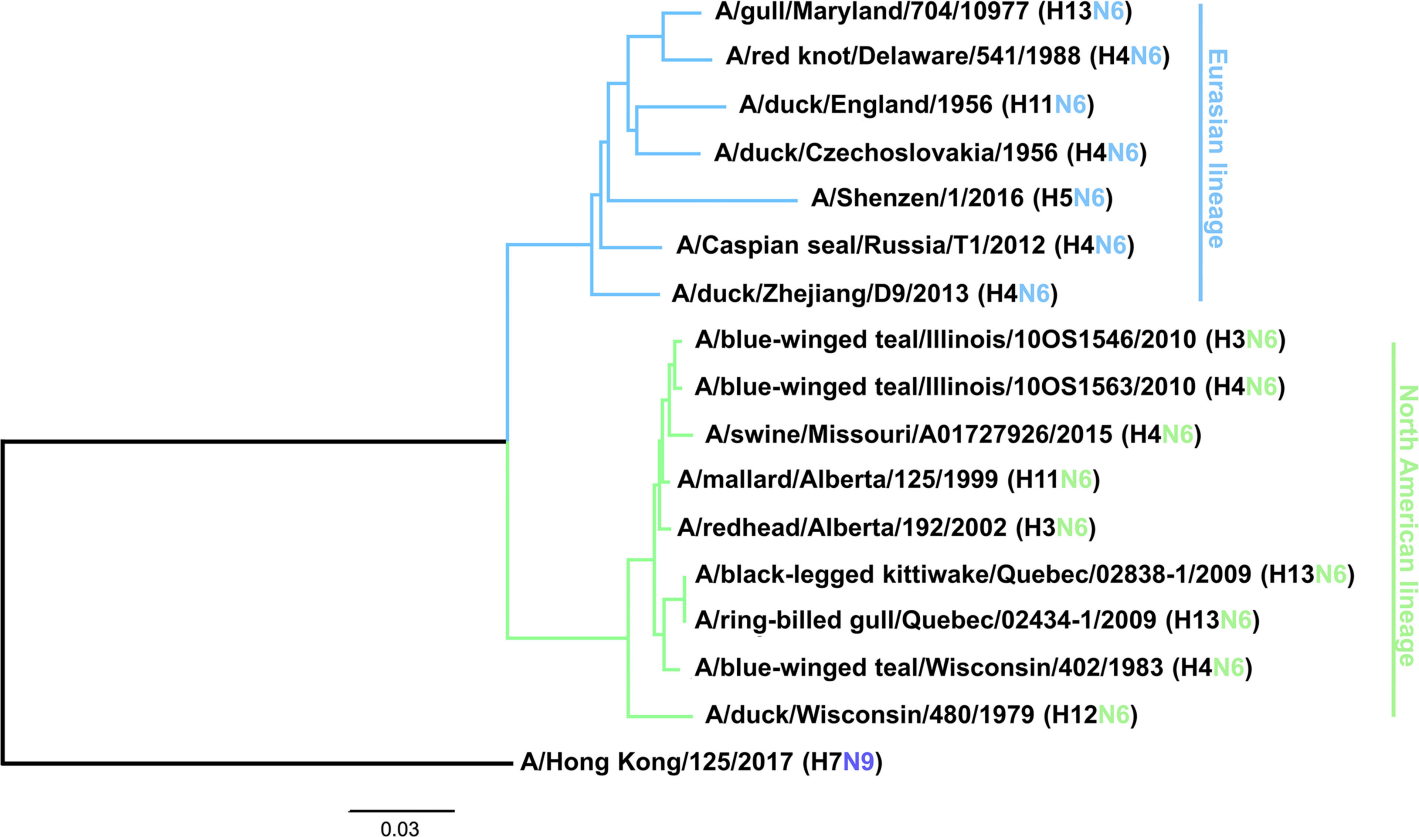
Phylogenetic tree of the N6 subtype based on amino acid sequences. A phylogenetic tree was constructed based on the amino acid sequence alignment of the various N6 NAs. N9 was used as an outgroup and the scale bar represents a 3% change in amino acid sequence. Eurasian and North American lineages are indicated. The tree was constructed in Clustal Omega and visualized in FigTree.

Viruses with N6 NA circulate in avian species in combination with all 16 HA subtypes, although H3N6 (28), H4N6 (22), H5N6 (29), H6N6 (30) and H13N6 (31) are most common. In addition, H3N6, H4N6, H5N6 and H6N6 have been isolated from swine (32–35), H4N6 viruses have been found in marine mammals (36) and H5N6 viruses have been found in cats (29). Avian H7N6 viruses have also been shown to be transmissible in guinea pigs (37). The N6 subtype can be further divided into two lineages, a predominantly Eurasian lineage (EAL) and a predominantly North American lineage (NAL) (**Figure 1**) (1), although isolates from North America sometimes fall into the Eurasian lineage and *vice versa*. Some N6 isolates have also been found to be resistant to NA inhibitors, which is a concern (38, 39). Another peculiarity that seems to be specific for N6 NAs is the ability of some strains to aid cleavage of HA in concert with thrombin-like proteases, as observed for an H7N6 virus isolate (40), which allows systemic replication of these viruses without the presence of a polybasic cleavage site of the HA.

With the emergence of H5NX viruses (41), highly pathogenic H5N6 viruses hosting a Eurasian N6 appeared in chickens in 2013 and have since 2015 also infected humans and have – according to the World Health Organization (WHO) as of February 4^th^ 2022 (42) - led to 66 laboratory confirmed infections with 29 fatalities (43–47). Therefore, H5N6 vaccine candidates are being prepared (e.g. (H5N6)-PR8-IDCDC-RG42A). While these vaccines are focused on inducing anti-HA antibodies, it would also be important to better understand the antigenicity of the N6 NA. Here, using murine hybridoma technology, we generated and characterized monoclonal antibodies (mAbs) to better understand breadth, functionality, epitopes and protective effect of the antibody response to N6 NA.

## Materials and Methods

### Viruses

The viruses A/duck/Wisconsin/480/1979 (H12N6; BEI Resources # NR-28616), A/redhead/Alberta/192/2002 (H3N6; BEI Resources # NR-45145), A/duck/England/1956 (H11N6; BEI Resources # NR-21660), A/gull/Maryland/704/1977 (H13N6; BEI Resources # NR-21663), A/black-legged kittiwake/Quebec/02838-1/2009 (H13N6; BEI Resources # NR-31217), A/ring-billed gull/Quebec/02434-1/2009 (H13N6; BEI Resources # NR-31218), A/mallard/Alberta/125/1999 (H11N6; BEI Resources # NR-45187), A/blue-winged teal/Illinois/10OS1546/2010 (H3N6; BEI Resources # NR-35981) and A/blue-winged teal/Wisconsin/402/1983 (H4N6; BEI Resources # NR-48971) were obtained from the Biodefense and Emerging Infections Research Resources Repository (BEI Resources). The viruses were propagated in 10 day old embryonated chicken eggs (Charles River Laboratories) at 37°C for 2 days. The viral titers were determined by conducting a standard plaque assay on Madin Darby canine kidney (MDCK) cells as described earlier (48). The viruses A/duck/Czechoslovakia/1956 (H4N6) and A/swine/Missouri/A01727926/2015 (H4N6) were rescued in the backbone of A/PR/8/34 as a 6:2 reassortant, containing the HA and NA segments of the original virus isolates (49, 50).

### Cells

MDCK cells were cultivated in Dulbecco’s Modified Eagle’s Medium (complete DMEM, Gibco) containing 1% penicillin/streptomycin (100 U/ml of penicillin, 100 µg/ml streptomycin, Gibco) antibiotic solution, 10% fetal bovine serum (FBS, Gibco) and 1% hydroxyethylpiperazine ethane sulfonic acid (HEPES). SP2/0-Ag14 myeloma cells were propagated in complete DMEM supplemented with 1% L-glutamine (Gibco).

High Five cells (BTI-TN-5B1-4, *Trichoplusia ni*) were grown in serum-free Express Five media (Gibco) containing 1% L-glutamine and 1% penicillin/streptomycin antibiotics mix. Sf9 cells (*Spodoptera frugiperda*), adapted from the cell line ATCC CRL-1711, were maintained in *Trichoplusia ni* medium – Fred Hink (TNM-FH, Gemini Bioproducts) supplemented with 1% penicillin/streptomycin antibiotics mix, 1% pluronic F-68 (Sigma-Aldrich) and 10% fetal bovine serum. In order to passage the baculoviruses, the media was switched to 3% TNM-FH (1% penicillin/streptomycin, 1% pluronic F-68, 3% FBS).

### Recombinant proteins

The recombinant N6 glycoproteins used in this study were expressed in insect cells using the baculovirus expression system. The globular head domain (aa85-385) of the respective N6 protein from the strains A/duck/Czechoslovakia/1956 (H4N6), A/Shenzen/1/2016 (H5N6), A/swine/Missouri/A01727926/2015 (H4N6), A/Caspian seal/Russia/T1/2012 (H4N6) and A/duck/Zhejiang/D9/2013 (H4N6) as well as for the N9 of A/Anhui/1/2013 (H7N9) were cloned into a baculovirus shuttle vector, containing an N-terminal signal peptide, followed by a hexahistidine purification tag, a VASP (vasodilator-stimulated phosphoprotein) tetramerization domain and a thrombin cleavage site (51). The baculoviruses were passaged in Sf9 cells to reach higher titers and were then used to infect High Five cells for protein expression. After three days of infection, the soluble proteins were purified from the supernatant, as previously described (52), and were then stored at -80°C for further usage.

### Enzyme-linked immunosorbent assay (ELISA)

Ninety-six well flat bottom plates (Immulon 4 HBX plates, ThermoFisher Scientific) were coated with 50 µl/well of 2 µg/ml recombinant protein in 1x coating buffer (Seracare) at 4°C overnight. The following day, the plate was washed three times with 100 µl/well of 0.1% Tween 20 (TPBS) to remove residual coating solution and then incubated with 100 µl/well of 3% milk dissolved in TPBS for 1h at room temperature to avoid non-specific binding. The blocking solution was removed and the primary antibody added at a starting concentration of 30 µg/ml in 1% milk/TPBS. The antibodies were serially diluted 1:3 and the plates were incubated for 1 hour at room temperature (RT). An anti-Lassa antibody (KL-AV-1A12 (53)) was included as a negative control and an anti-histidine antibody as a positive control. The plate was then washed three times with 100 µl/well TPBS and an anti-mouse secondary antibody (anti-mouse IgG H&L antibody peroxidase conjugated, Rockland) diluted 1:3000 in 1% milk/TBPS was added for 1h at RT. The plate was washed three times and 100 µl/well of SigmaFast o-Phenylenediamine dihydrochloride (OPD) developing solution (Sigma Aldrich) were added. The reaction was stopped after 10 min incubation at RT with 50 µl/well of 3M hydrochloric acid (HCl). The plate was read with a Synergy H1 hybrid multimode microplate reader (BioTek) at an optical density of 490 nm. The data was analyzed by using GraphPad Prism 7 software. The antibodies were tested in duplicates and the endpoint titers were defined as the final concentration at which the antibody binding signal was higher than 3 standard deviations above the average of the blank wells.

### Generation of monoclonal N6-antibodies

Mouse mAbs were produced using hybridoma technology, as previously described (54, 55). Briefly, female 6-8 week old BALB/c mice (The Jackson Laboratory; n=5) were immunized *via* the intraperitoneal route with A/swine/Missouri/A01727926/2015 N6 antigen in 100 µl PBS, the preparation was used 21 days later for an intranasal immunization. Three weeks later, the mice were boosted with A/Shenzhen/1/2016 N6 antigen in a volume of 50 µl. After three weeks, one mouse received a final boost intraperitoneally with 100 µg of N6 antigen from A/duck/Zhejiang/D9/2013 (H4N6) adjuvanted with 10 µg of poly (I:C) (*In vivo*gen). Three days after the final boost, the mouse was euthanized and the spleen removed. The spleen was washed with PBS and then flushed with serum-free DMEM (with 1% penicillin/streptomycin) to obtain the splenocytes. The splenocytes were fused with SP2/0-Ag14 myeloma cells in a ratio of 5:1 using pre-warmed polyethylene glycol (PEG; Sigma-Aldrich). The cells were grown on semi-solid selection & cloning medium with hypoxanthine-aminopterin-thymidine (HAT; Molecular Devices) for 10 days and were then expanded to a 96-well cell culture plate.

To obtain antibodies specific for N6, the supernatant of the individual clones was screened *via* ELISA against recombinant NA of A/duck/Zhejiang/D9/2013 (H4N6). Reactive clones were tested using the Pierce rapid antibody isotyping kit (Life Technologies). Only IgG heavy-chain expressing clones were continuously expanded. The selected hybridoma clones were first expanded in Clonacell-HY Medium E and then switched to Hybridoma SFM media (Gibco) supplemented with 1% penicillin/streptomycin. The antibodies were then purified as previously described (13). To make sure the obtained mAbs are not confused with other mAbs made in the laboratory, we use the nomenclature ‘KL-N6-XXX’ where ‘KL’ stands for ‘Krammer Laboratory’, ‘N6’ for the target and the ‘XXX’ stands for a combination of letters and numbers referring to the position on a screening plate. The full names can be found in **Table 1** but throughout the text only the ‘XXX’ is used for simplicity.

**Table 1:** Antibody isotypes.

Anti-N6 mAb	Isotype
KL-N6-1A5 (1A5)	IgG2a
KL-N6-1D4 (1D4)	IgG2a
KL-N6-1G9 (1G9)	IgG2a
KL-N6-2E8 (2E8)	IgG2a
KL-N6-3B5 (3B5)	IgG2a
KL-N6-3C6 (3C6)	IgG2a
KL-N6-3C7 (3C7)	IgG2a
KL-N6-3D5 (3D5)	IgG2a
KL-N6-3E1 (3E1)	IgG2a
KL-N6-(3F10)	IgG2a

### Immunofluorescence assay

MDCK cells were seeded in a sterile 96-well cell culture plate at a density of 25,000 cells/well using complete DMEM. The next day, cells were infected with a multiplicity of infection (MOI) of 3.0 for 16h using serum free minimal essential media (MEM, Gibco), supplemented with penicillin/streptomycin, HEPES (Gibco), glutamine (200mM 1-glutamine, Gibco) and sodium bicarbonate (sodium bicarbonate 7.5% solution, Gibco). The cells were fixed with 3.7% paraformaldehyde (PFA)/PBS for 1h at RT. The plate was the blocked for 1h with 3%milk/PBS. The antibodies were diluted to 30 µg/ml in 1% milk/PBS and incubated for 1h at RT. An anti-Lassa antibody [(KL-AV-1A12 (53)] was included as a negative control and CR9114 (56), a broadly cross-reactive HA antibody, as a positive control. The plate was washed three times with 1xPBS and then incubated with a fluorescence goat anti-mouse IgG heavy plus light chain (H+L)–Alexa Fluor 488 antibody (Abcam), which was diluted 1:1000 in 1% milk/PBS. The cells were washed with PBS and kept in PBS to avoid drying out during microscopy (Olympus IX-70).

### Antibody-dependent cellular cytotoxicity reporter assay

To assess potential ADCC activity of the N6 mAbs the ADCC reporter bioassay kit from Promega was used (57). MDCK cells (25,000 cells/well) were seeded in a white, flat bottom 96- well cell culture plate (Corning). The following day the cells were washed with PBS and then infected with an MOI of 1 with the respective virus at 37°C for 16h. On the following day, antibody dilutions were prepared using a starting concentration of 100 µg/ml. The antibodies were serially diluted 1:3 and then added in duplicates to the cells. The human derived monoclonal antibody CR9114 (56) was included as a positive control and an irrelevant anti-Lassa virus antibody [KL-AV-1A12 (53)] was used as a negative control. Next, 75,000 effector cells/well were added to the plate and incubated for 6 h at 37°C. The luciferase substrate was added in the dark and the luminescence activity measured after 5 minutes using a Synergy Hybrid Reader (BioTek). The antibodies were run in duplicates and the data was analyzed using GraphPad Prism 7.

### NA inhibition assay

NA inhibition assays were performed as described in detail earlier (12, 55). In brief, 96-well flat bottom Immulon 4 HBX plates (ThermoFisher Scientific) were coated with 150 µl/well of fetuin (Sigma Aldrich) at a concentration of 50 µg/ml overnight at 4°C. The following day, the N6 mAbs were serially diluted 1:3 in PBS with a starting concentration of 30 µg/ml. The respective viruses were diluted in PBS at 2x the 50% effective concentration (EC_50_), which was determined in an NA- assay previously. The virus (75 µl/well) was then added to the mAb dilution plate and the virus/mAb mixture was incubated for 1h 45 min shaking at RT. During this time, the fetuin coated plates were washed 6x with PBST and then blocked with 5% bovine serum albumin (BSA)/PBS for at least 1h at RT. After 1 hour, the fetuin plates were washed and 100 µl of the virus/antibody mixture were added to plates and incubated for 2h at 37°C. The plates were washed and incubated with 5µg/100µl/well of horseradish peroxidase labeled peanut agglutinin (PNA) for 1h 45 min in the dark. The plates were then washed and developed with 100 µl/well Sigmafast OPD solution (Sigma Aldrich). After 7 minutes of incubation in the dark, the reaction was stopped by adding 50 µl/well of 3M HCl and the plates read at an absorbance of 490 nm using a Synergy H1 hybrid multimode microplate reader (BioTek). The antibodies were run in duplicates and the IC_50_ values were calculated using GraphPad Prism 7.

### Plaque reduction neutralization assay

Plaque reduction neutralization assays were performed on MDCK cells (300,000 cells/well) which were seeded in a sterile 12-well plate the day before. The following day, the mAbs were serially diluted 1:3 in 1x MEM and 50 µl of A/duck/Czechoslovakia/1956 (H4N6, 1000 PFU/ml) were added to each dilution and incubated shaking at RT for 1h. The cells were washed with PBS and the antibody-virus mixture was added for 1h at 37°C. The mixture was then aspirated and the cells overlaid with agar consisting of minimal essential medium (2xMEM), 2% Oxoid agar, 1% diethylaminoethyl (DEAE) dextran, tosyl phenylalanyl chloromethyl ketone (TPCK)-treated trypsin as well as the respective antibody. The plates were incubated at 37°C for 2 days and the cells were afterwards fixed with 3.7% PFA in PBS. The plaques were then visualized by immunostaining. Briefly, the agar-overlay was removed and the plates blocked with 3% milk/PBS for 1h at RT. Afterwards, an antibody-cocktail made out of all ten N6-antibodies diluted 1:3000 in milk/PBS was added and incubated for 2h at RT. The plates were washed and incubated with secondary antibody (Anti-mouse IgG (H&L) Antibody Peroxidase Conjugated, Rockland) diluted 1:3000 in 1% milk/PBS for 1 h. The plates were washed and developed using Trueblue reagent (KPL). The plaque number as well as the plaque size was determined for each antibody dilution and the percent inhibition determined based on a no-antibody control. The antibodies were run in duplicates and the data was analyzed using GraphPhad Prism 7 (55, 58).

### Evaluation of the prophylactic efficacy in mice

To test the prophylactic efficacy of the N6 antibodies, female 6-8 week old DBA/2J mice (The Jackson Laboratory) received an antibody dose of 5mg/kg intraperitoneal (n=5 per group). An irrelevant anti-Lassa antibody [KL-AV-1A12 (53)] was given to one of the groups as negative control. Two hours after antibody administration, the mice were anesthetized and intranasally challenged with 5x mLD_50_ (mouse lethal dose, 50%) of A/duck/Czechoslovakia/1956 (H4N6, A/Puerto Rico/8/34 reassortant). Weight loss was monitored over 14 days and any mouse which lost more than 25% of its initial body weight was euthanized and scored dead. Survival and weight loss data were analyzed by using GraphPad Prism 7. All antibodies were tested at the same time in one animal study. Animal procedures were performed in accordance with protocols approved by the Icahn School of Medicine at Mount Sinai Institutional Animal Care and Use Committee (IACUC).

### Escape mutants

MDCKs cells were plated at a density of 1,000,000 cells/well in a 6- well plate the day prior. The next day, cells were washed with PBS and infected with an multiplicity of infection (MOI) of 1 with the virus A/duck/Czechoslovakia/1956 (H4N6) in 1xMEM as well as 1 ug/ml TPCK-treated trypsin. The respective mAbs were added at a concentration of 1xIC_50_ to the virus mixture and the cells were then incubated for 2 days at 37°C. The cell supernatant was collected and used to continue the passaging of the virus. The concentration of the mAbs was doubled with every passage. This procedure was repeated until an IC_50_ concentration of 128 was reached. The presence of the virus within the samples was confirmed by performing an immunostaining of the cell layer after every passage. The same virus was passaged in parallel completely without mAbs as well as together with an irrelevant antibody [anti-Lassa KL-AV-1A12 (53)], to confirm that no random mutations were acquired over time.

Once the last passage was reached, the viruses were plaque purified and injected, together with the respective mAb, in 10-day old embryonated chicken eggs, to propagate the virus. The eggs were incubated for 2 days at 37°C. RNA was extracted from the allantoic fluid by using the Direct-zol RNA isolation kit from Zymo research. The purified RNA was used to deep-sequence the escape viruses.

### Visualization of protein structure

The tetrameric head domain of the NA protein of A/duck/England/1/1956 (H4N6, PDB 1V0Z) was visualized using PyMOL. The structure is shown in top down, bottom up and side view to highlight the individual mutations identified through epitope mapping. One of the monomers has been highlighted in light grey.

## Results

### Characterization of antibody binding breadth

**Figure 2:**
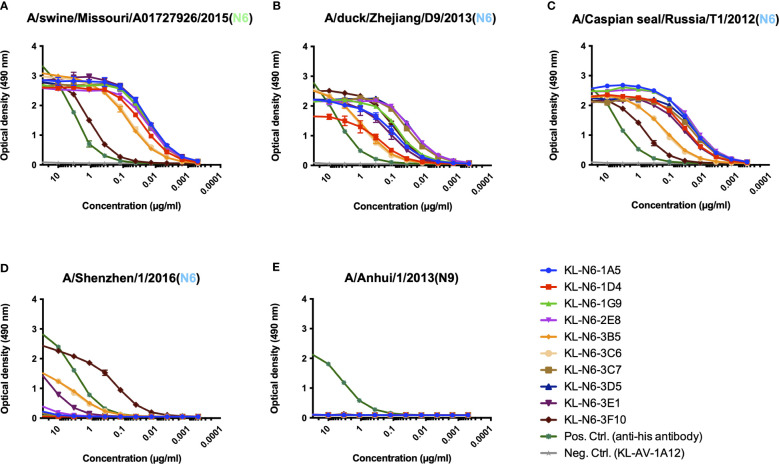
Murine N6 NA mAbs are highly specific to the N6 subtype and show broad cross-reactivity to both Eurasian and North American lineage NAs in ELISA. Binding of murine N6 NA mAbs to A/swine/Missouri/A01727926/2015 **(A)**, A/duck/Zhejiang/D9/2013 **(B)** and A/Caspian seal/Russia/T1/2012 **(C)** and A/Shenzhen/1/2016 **(D)** N6 NA recombinant protein. Binding of murine N6 NA mAbs to A/Anhui/1/2013 **(E)** N9 NA recombinant protein. None of the antibodies showed reactivity against recombinant N9 protein, which is the closest related NA to N6. An anti-his antibody was used as a positive control and an irrelevant anti-Lassa antibody (KL-AV-1A12) was used as a negative control. Error bars shown represent the standard deviation.

**Figure 3:**
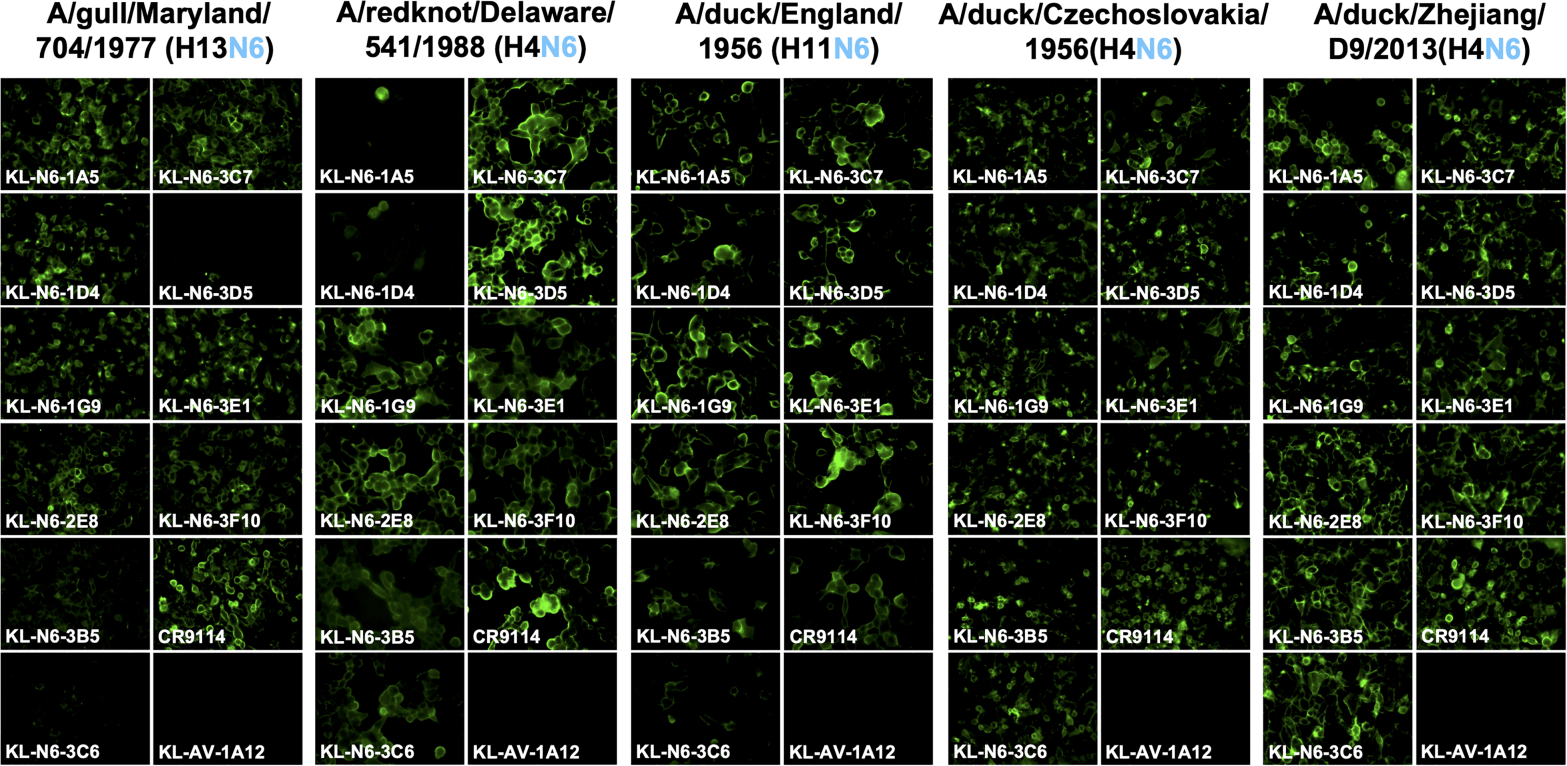
Murine N6 NA mAb binding of Eurasian lineage virus infected MDCK cells. MDCK cells were infected at an MOI of 3 with Eurasian lineage viruses for 16h. The infected cells were then fixed and the N6 mAbs were added at a concentration of 30 ug/mL. mAb CR9114 was used as positive control and an anti-Lassa antibody KL-AV-1A12 was used as negative control.

**Figure 4:**
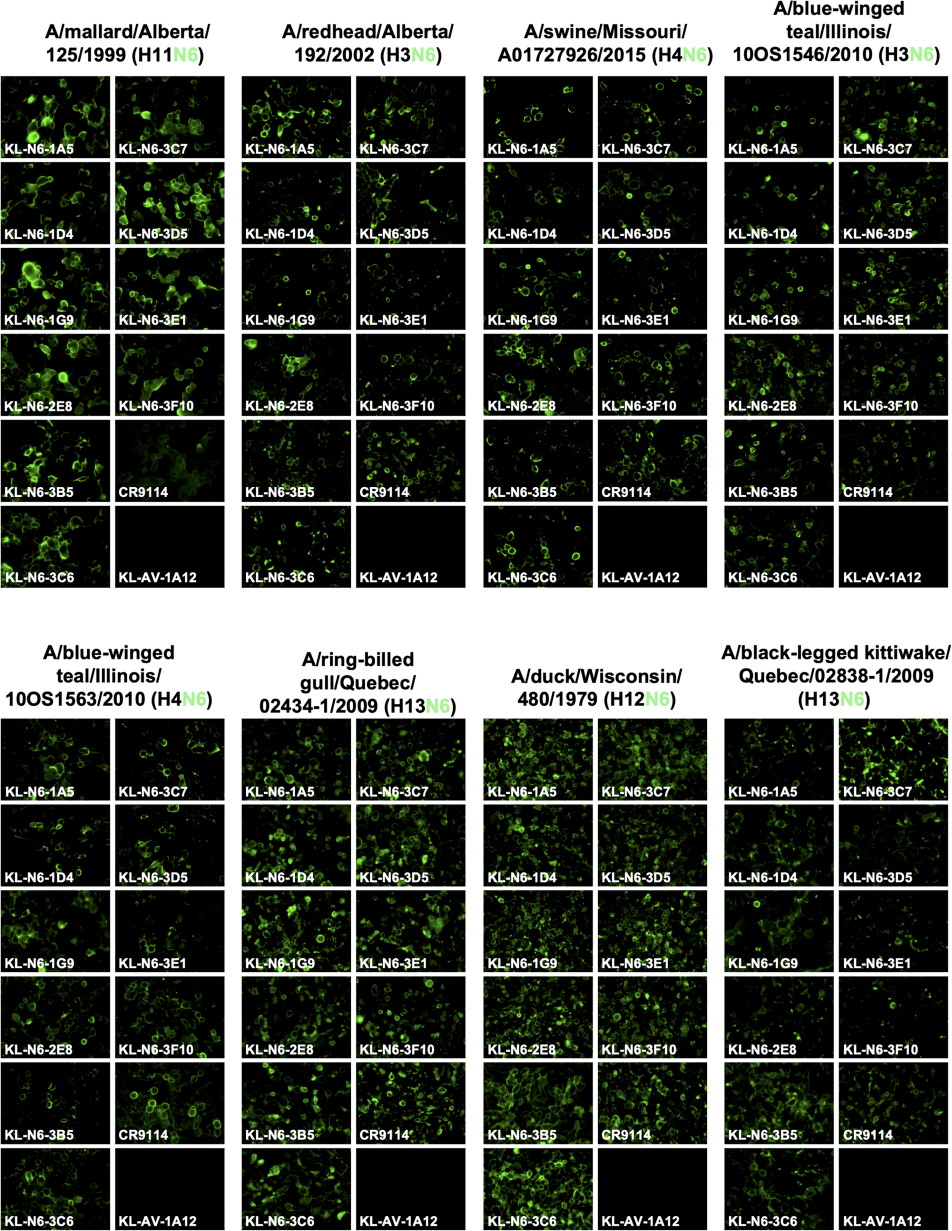
Murine N6 NA mAb binding of North American lineage virus infected MDCK cells. MDCK cells were infected at an MOI of 3 with North American lineage viruses for 16h. The infected cells were then fixed and the N6 mAbs were added at a concentration of 30 ug/mL. The N6 mAbs show broad cross reactivity within the subset of North American lineage viruses. mAb CR9114 was used as positive control and an anti-Lassa antibody KL-AV-1A12 as negative control.

Ten mAbs with strong reactivity to the N6 NA of A/swine/Missouri/A01727926/2015 were identified as a result of the hybridoma fusion (**Table 1**). We wanted to characterize the binding breadth of the ten mAbs. To do this, we recombinantly expressed the N6 of A/swine/Missouri/A01727926/2015 (H4N6, NAL), A/Shenzhen/1/2016 (H5N6, EAL), A/Caspian seal/Russia/T1/2012 (H4N6, EAL) and A/duck/Zhejiang/D9/2013 (H4N6, EAL) as soluble tetramers. These proteins were used as substrates for ELISAs. The N9 protein of A/Anhui/1/2013 (H7N9) was used as a negative control to ensure that mAbs were specific to the N6. All mAbs, except 3F10, displayed strong reactivity to the N6 NA of A/swine/Missouri/A01727926/2015 (NAL, **Figure 2A**). MAb 3F10, while still binding, had a higher endpoint titer (0.04115 µg/ml) compared to the other nine mAbs. Binding to the N6 NA of A/duck/Zhejiang/D9/2013 (EAL) and A/Caspian seal/Russia/T1/2012 (EAL) was more variable, but all mAbs reacted well in an ELISA and better than the positive control (**Figures 2B, C**). Interestingly, binding to the recombinant N6 NA of A/Shenzhen/1/2016 (EAL) was low for most mAbs, and only mAb 3F10 showed decent binding with an ELISA endpoint titer of 0.00152 µg/ml. Lower binding activity was observed for 3B5, 3C5 and 3E1 (**Figure 2D**). None of the mAbs bound to recombinant N9 NA which shows that antibodies were specific to the N6 (**Figure 2E**) (**Table 2**). In addition to the ELISA, we also performed an immunofluorescence assay (IF) of MDCK cells infected with wild type isolates and re-assortant viruses from both lineages. In this assay, a majority of mAbs bound to all six tested EAL viruses (**Figure 3**). Similar to the EAL lineage, all mAbs bound to cells infected by all eight NAL viruses in this assay (**Figure 4**). In summary, many of the isolated mAbs show broad binding activity to EAL and NAL N6 NAs.

**Table 2:** Quantitative assay results.

Virus	KL-N6-1A5	KL-N6-1D4	KL-N6-1G9	KL-N6-2E8	KL-N6-3B5	KL-N6-3C6	KL-N6-3C7	KL-N6-3D5	KL-N6-3E1	KL-N6-3F10	Assay-Type
A/duck/Zhejiang/D9/2013 (H4N6)	0.0015	0.0030	0.0015	0.00051	0.0046	0.0046	0.00051	0.00051	0.0030	0.0015	ELISA EPT
A/Caspian seal/Russia/T1/2012 (H4N6)	0.00051	0.00051	0.00051	0.00051	0.0015	0.0015	0.00051	0.00051	0.00051	0.014	ELISA EPT
A/swine/Missouri/A01727926/2015 (H4N6)	0.00051	0.00051	0.00051	0.00051	0.0015	0.0015	0.00051	0.00051	0.00051	0.041	ELISA EPT
	0.042	0.27	0.042	0.024	6.96	7.63	0.027	0.021	0.31	2.44	NAI IC_50_
	500.5	467.1	429.2	272.3	682.1	697.6	433.8	343.2	505.9	114.2	ADCC AUC
A/duck/Czechoslovakia/1956 (H4N6)	0.62	2.42	0.51	0.029	14.84	0.027	32.8	0.023	3.12	1.89	NAI IC_50_
	1.55	3.42	0.45	3.47	7.86	ND	ND	3.81	3.45	ND	PRNT IC_50_
	209.6	155.0	147.7	203.4	302.3	338.6	253.3	196.2	147.2	158.4	ADCC AUC
A/Shenzhen/1/2016 (H5N6)	3.33	30	20	1.11	0.041	0.041	3.33	3.33	0.37	0.0015	ELISA EPT
A/black-legged kittiwake/Quebec/02838-1/2009 (H13N6)	0.20	0.70	0.26	0.030	14.93	12.13	0.024	0.010	0.27	0.13	NAI IC_50_
	249.8	202.2	233.5	221.5	239.5	205.5	249.1	246.3	287.0	204.1	ADCC AUC

ND, not detected; EPT, endpoint titer; AUC, area under the curve.

### *In vitro* functional characterization of mAbs

**Figure 5:**
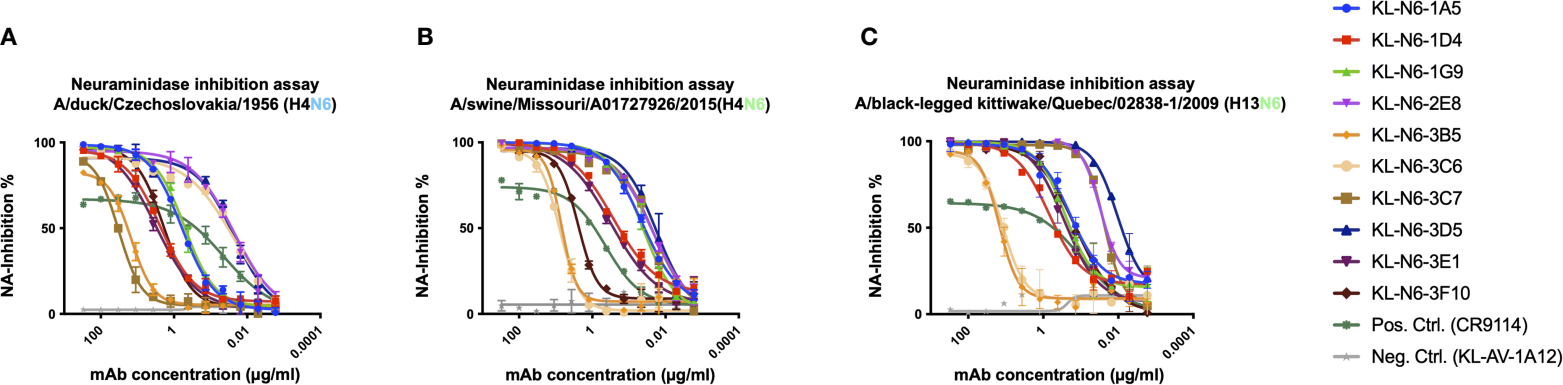
N6 mAbs strongly inhibit NA enzymatic activity. N6 NA mAbs were tested for inhibition of A/duck/Czechoslovakia/1956 (H4N6) **(A)**, A/swine/Missouri/A01727926/2015 (H4N6) **(B)**, and A/black-legged kittiwake/Quebec/02838-1/2009 (H13N6) **(C)**. The broad HA stalk antibody CR9114 was used as a positive control as it exhibits NA inhibition based on steric hindrance and an irrelevant anti-Lassa antibody KL-AV-1A12 was used as negative control. Error bars shown represent the standard deviation.

Next, we wanted to test the *in vitro* functionality of the isolated mAbs in NA inhibition (NI), plaque reduction neutralization (PRNT) and antibody-dependent cellular cytotoxicity (ADCC) reporter assays. NI was measured against A/duck/Czechoslovakia/1956 (EAL, H4N6), A/swine/Missouri/A01727926/2015 (NAL, H4N6) and A/black-legged kittiwake/Quebec/02838-1/2009 (NAL, H13N6). MAbs 2E8, 3C6 and 3D5 showed the strongest inhibition of A/duck/Czechoslovakia/1956 followed by 1G9, 1A5, 3F10, 1D4, 3E1, 3B5 and finally 3C6 (**Figure 5A**). The order was similar for A/swine/Missouri/A01727926/2015 except that 3F10 was only slightly better than 3B5 and finally 3C6 (**Figure 5B**). For A/black-legged kittiwake/Quebec/02838-1/2009 (NAL, H13N6) the pattern looked similar to A/Swine/Missouri/A01727926/2015, however in this case 3F10 performed better than 1D4, 3B5 and 3C6 (**Figure 5C**). Of note, HA stalk-reactive antibody CR9114 (56, 59), which can inhibit NA through steric hindrance (60), was used as positive control.

**Figure 6:**
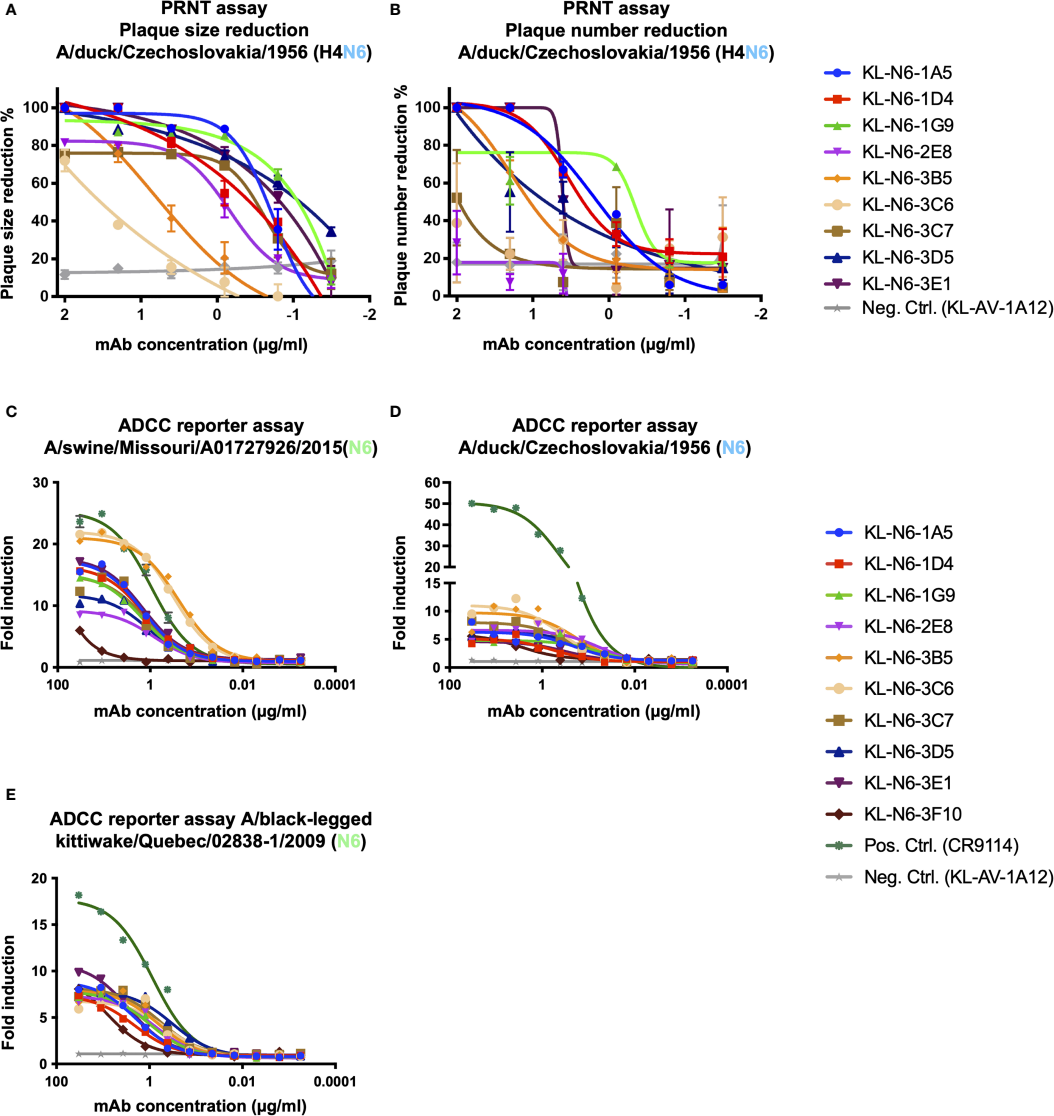
*In vitro* functional characterization of the N6 mAbs. **(A)** Plaque size and **(B)** number reduction measured *via* plaque reduction neutralization assay. **(C–E)** ADCC reporter activity of mAbs using infected MDCK cells as the substrate. Luminescence was measured at the end of the assay to assess ADCC activity. The HA stalk antibody CR9114 was used as a positive control and an irrelevant anti-Lassa antibody KL-AV-1A12 as negative control. Error bars shown represent the standard deviation.

Using A/duck/Czechoslovakia/1956 (H4N6), we also performed plaque reduction neutralization assays (PRNTs) to measure both reduction of plaque size (a common feature of anti-NA mAbs) and reduction of plaque numbers (usually referred to as neutralization). All mAbs reduced plaque size to various degrees, mostly in accordance with their NI activity (**Figure 6A**). Plaque number was only reduced effectively by mAbs 1A5, 1D4, 1G9, 3B5, 3D5 and 3E1 (**Figure 6B**). Interestingly, these results are not in concordance with the NI activity suggesting that neutralization requires a different mechanism than NI activity for these mAbs.

ADCC reporter activity was also measured since it has been shown to be important for the protective effect of some NA mAbs. All antibodies except 3F10 exhibited reasonable but variable ADCC reporter activity against A/swine/Missouri/A01727926/2015. Especially 3B5 and 3C6 showed outstanding activity that was higher than that of the positive control, stalk mAbs CR9114 which is known for its ADCC activity (**Figure 6C**). Interestingly, the ADCC reporter activity of the mAbs was much lower against cells infected with A/duck/Czechoslovakia/1956 (**Figure 6D**) or A/black-legged kittiwake/Quebec/02838-1/2009 (**Figure 6E**, **Table 2**).

To summarize the *in vitro* characterization, all mAbs inhibited NA activity in *in vitro* assays, albeit at various degrees. Activity in ADCC reporter assays was variable and was strongly influenced by the virus tested.

### Epitope mapping *via* escape mutant generation

**Figure 7:**
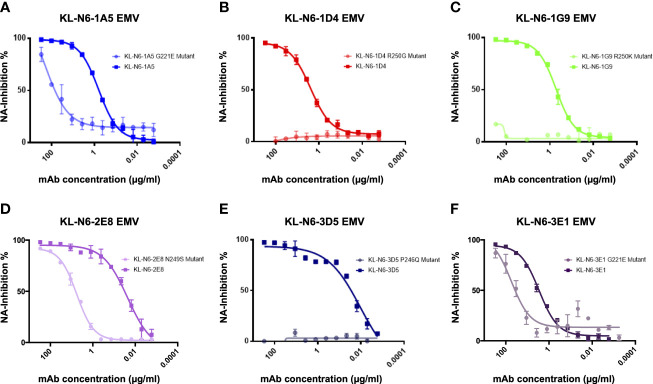
Effect on NA inhibition of N6 mAbs against escape mutants. **(A, D, F)** Antibodies KL-N6-1A5, KL-N6-2E8 and KL-N6-3E1 show decreased neuraminidase inhibition activity to the respective escape mutant viruses (EMVs). EMV 1A5 and EMV 3E1 have the same mutation at position 221 (G221E), whereas EMV 2E8 has a mutation at position 249 (N249S). **(B, C, E)** KL-N6-1D4, KL-N6-1G9 and KL-N6-3D5 display a complete loss in neuraminidase inhibition activity against the respective EMVs. EMV 1D4 and EMV 1G9 have a mutation at the same position at aa 250 (R250G, R250K). EMV 3D5 has a mutation at position 246 (P246Q). Error bars shown represent the standard deviation.

**Figure 8:**
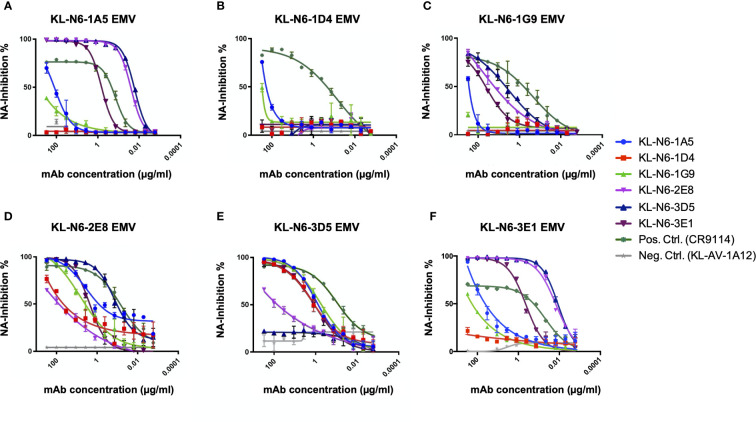
Cross NA inhibition testing of EMVs. **(A)** 1A5 EMV (G221E) strongly reduces NI of KL-N6-1D4 and KL-N6-1G9, whereas the inhibition capability of KL-N6-2E8 and KL-N6-3D5 fully remains. **(B)** 1D4 EMV (R250G) abolishes NI of all mAbs to a large degree. **(C)** 1G9 EMV (R250K) causes total loss of inhibition by KL-N6-1A5 and KL-N6-1D4. **(D)** 2E8 EMV (N249S) does not impact NI inhibition significally. **(E)** 3D5 EMV (P249Q) leads to a decreased NI through KL-N6-2E8 but has less impact on the remaining mAbs. **(F)** 3E1 EMV (G221) shows a similar pattern as 1A5 EMV. As a positive control the broad HA stalk antibody CR9114 was used and as a negative control KL-AV-1A12 was included. Error bars shown represent the standard deviation.

**Figure 9:**
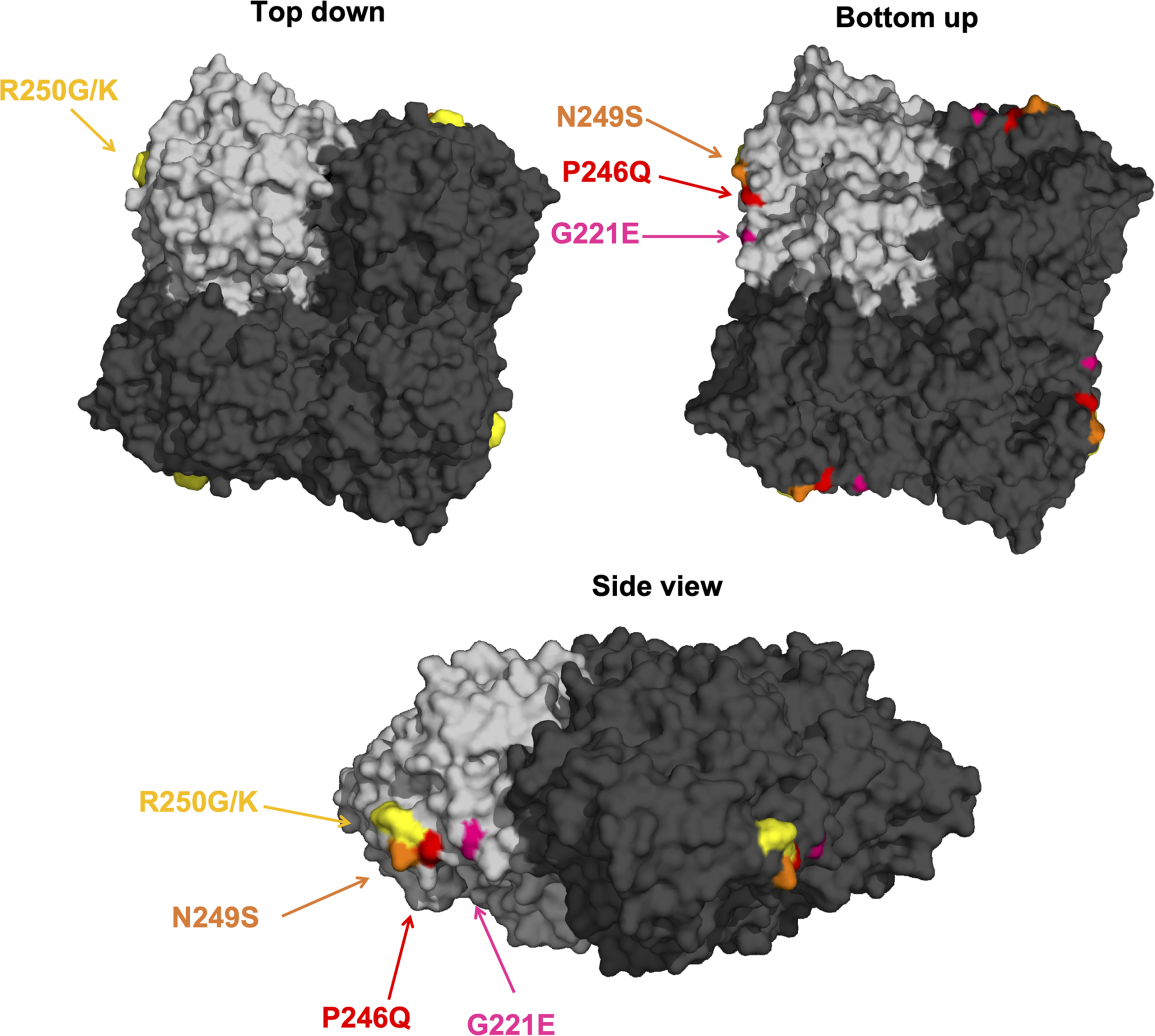
Visualization of N6 NA mAb escape mutations. The tetrameric protein of A/duck/England/1/1956 (H4N6) is shown in top down, bottom up and side view with one of the monomers highlighted in light grey. The individual mutations are highlighted in different colors and are in close proximity on the lateral surface of the NA. The graphic is based on PDB# 1V0Z.

To learn more about the epitopes of the generated mAbs, we generated escape mutant viruses (EMVs) by subjecting A/duck/Czechoslovakia/1956 to increasing antibody pressure by passaging the virus with increasing amounts of antibody as described before (20, 21). An irrelevant mAb directed against the Lassa virus glycoprotein was included as control (53). We succeeded in generating six different EMVs. The EMV selected by 1A5 showed a strong reduction in NI compared to wild type. This EMV carried a G221E mutation on the lateral side of the NA (**Figure 7**). Similar phenotypes were seen for the 2E8 (N249S) and 3E1 (G221E) EMVs. For the 1D4 (R250G), 1G9 (R250K) and 3D5 (P246Q) EMVs, a complete loss of NI was observed. When the six mAbs were then cross-tested with the six EMVs, we found that the 1A5 EMV (G221E) also strongly reduced NI of 1D4 and 1G9 while 2E8 and 3D5 still showed good NI (**Figure 8**). The 1D4 (R250G) mutation abolished NI of all mAbs to a large degree. The 1G9 EMV (R250K) had a lower impact and only caused loss of inhibition by 1A5 and 1D4 in addition to 1G9, likely owing this to its conserved nature of the amino acid change. For the 2E8 EMV (N249S) all mAbs retained some NI activity. The 3D5 mutation (P249Q)) had an impact on 2E8 and itself, but less so on the other mAbs. Finally, the 3E1 EMV, which carries the same G221E mutation in the NA as the 1A5 EMV, showed a similar pattern as the 1A5 EMV. However, the impact on NI by 3E1 was stronger, potentially due to additional mutations in non-NA genes of this EMV which could have contributed to 3E1 specific escape. The interdependence of the EMV/mAb combinations makes sense as all escape mutations map to the same patch on the NA (**Figure 9**). Of note, it is certainly interesting, that all of the mapped mAbs, a majority of the ones isolated, seemed to target the same patch on NA.

### *In vivo* protective effect of mAbs

**Figure 10:**
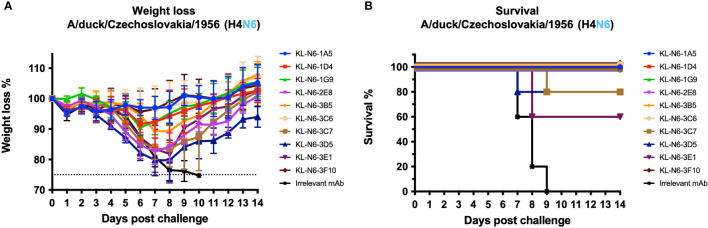
*In vivo* challenge study after prophylactic mAb treatment. N6 NA mAbs were administered to mice at a concentration of 5 mg/kg two hours prior to challenge. An irrelevant anti-Lassa antibody (KL-AV-1A12) was given as a negative control. Two hours after mAb administration, mice were challenged with 5xLD_50_ of A/duck/Czechoslovakia/1956 (H4N6). Morbidity **(A)** and mortality **(B)** following A/duck/Czechoslovakia/1956 (H4N6), A/Puerto Rico/8/34 reassortant challenge. Error bars shown represent the standard deviation.

Lastly, we wanted to determine the protective effect of the isolated mAbs against viral challenge in a mouse model. DBA/2J mice were administered antibody *via* the intraperitoneal route two at 5 mg/kg of mAb and then challenged 2 hours later intranasally with 5 times the 50% lethal dose (mLD50) of A/duck/Czechoslovakia/1956 (H4N6, EAL). Weight loss and survival was then monitored for 14 days. Protection from A/duck/Czechoslovakia/1956 was variable, with almost no weight loss and no mortality observed with 1A5, 3C6 and 3F10 (**Figures 10A,B**). 1D4, 1G9, 2E8 and 3B5 were also protective but mice experienced a transient body weight loss (approximately 10%). 3C7, 3D5 and 3E1 were less protective against weight loss and for 3C7 and 3E1 20% and 40% of the animals succumbed to infection respectively. In summary, while all antibodies seemed to provide a degree of protection at the tested dose, there was a wide range from complete protection from any weight loss to partial protection against mortality.

## Discussion

In this study, we are shedding first light on antigenicity of the N6 NA. As expected, we found significant cross-reactivity of the isolated mAbs. This could be due to our immunization regimen which might have favored cross-reactive epitopes but it may also be a consequence of the low level of antigenic drift for most avian derived N6 NAs. The characterized mAbs showed strong NI activity against isolates from the Eurasian and the North American lineage and protected – to various degrees – against challenge with a historic Eurasian lineage H4N6 strain. We also mapped the putative epitopes of six of the mAbs through generating escape mutants. Interestingly, all escape mutations were in close proximity on the lateral surface of the NA. This patch is reminiscent of the binding side of CD6 and other N1 specific and B NA specific antibodies that may span two monomers (13, 18, 61). This focus on one patch of the NA surface may be a consequence of our immunization regimen or may be specific to the N6 NA. While a similar area is also targeted by N1, N2 and B NA directed antibodies, many other sites, including the enzymatic site, are targeted for these subtypes as well (9, 11, 13, 14, 16–21). Interestingly, the six mapped antibodies, which focus on the lateral surface show very different activities *in vivo* ranging from complete protection from weight loss to partial protection from mortality. Of note, there is some agreement but also discordance between *in vivo* protection and *in vitro* activities for the respective mAbs in general. Of the three mAbs performing best *in vivo*, one (3C6) had strong and two had moderate (1A5 and 3F10) NI activity. 1A5 had strong activity in the PRNT assay, but 3C6 did not have much activity here. However, 3C6 had outstanding activity in the ADCC reporter assay where 1A5 and 3F10 had only moderate activity against the challenge virus. It therefore appears that different *in vitro* activities are associated with *in vivo* protection. Of note, some of the antibodies which did less well *in vivo* were amongst the ones which performed best in the NI assay. While our work provides first insights into the antigenicity of the N6 NA, much more work is needed to better understand N6 NA immunogenicity, epitopes and antigenic drift.

## Data availability statement

The raw data supporting the conclusions of this article will be made available by the authors, without undue reservation.

## Ethics statement

All animal procedures were performed in accordance with protocols approved by the Icahn School of Medicine at Mount Sinai Institutional Animal Care and Use Committee (IACUC)

## Author contributions

SS, FA and JC performed experiments. SS and FK analyzed data. SS and FK wrote the manuscript. All authors read and edited the manuscript.

## Funding

This work was supported in part by the National Institute of Allergy and Infectious Disease (NIAID) Centers of Excellence for Influenza Research and Surveillance (CEIRS) contract (HHSN272201400008C) and unrestricted institutional funds.

## Acknowledgments

We thank Parnavi Desai for excellent technical support.

## Conflict of interest

FK is listed as inventor on patent applications regarding NA-based vaccines and NA-based therapeutic mAbs. These patent applications have been filed by the Icahn School of Medicine at Mount Sinai and Washington University. Furthermore, FK is consulting for Pfizer, Merck, Third Rock Ventures and Avimex, and had past research support from Pfizer and GSK and current support from Toscana Life Sciences (TLS) Foundation.

The remaining authors declare that the research was conducted in the absence of any commercial or financial relationships that could be construed as a potential conflict of interest.

## Publisher’s Note

All claims expressed in this article are solely those of the authors and do not necessarily represent those of their affiliated organizations, or those of the publisher, the editors and the reviewers. Any product that may be evaluated in this article, or claim that may be made by its manufacturer, is not guaranteed or endorsed by the publisher.
